# Infective endocarditis uncovered a very rare congenital heart disease

**DOI:** 10.1002/ccr3.4216

**Published:** 2021-06-17

**Authors:** Fatemeh Jafari, Seyed‐Alireza Mirabdollahi, Shirin Jafari, Arezoo Saberi, Khadije Mohammadi

**Affiliations:** ^1^ Cardiovascular Research Center Kerman University of Medical Sciences Kerman Iran; ^2^ Department of Orthopedics School of Medicine Kerman University of Medical Sciences Kerman Iran

**Keywords:** aortic valve, congenital heart defect, dissection, infective endocarditis, ventricular septal defect

## Abstract

In this article, we introduced a case of rare congenital anomalies that was asymptomatic until adulthood and was complicated by infective endocarditis and dissection of aortic valve leaflet.

## Graphical abstract

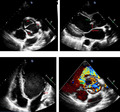

## 1 INTRODUCTION

Congenital heart disease (CHD) is one of important predisposing factors for infective endocarditis; here, we described a complicated case of infective endocarditis with multiple septic emboli and dissected right coronary cusp in context of very rare congenital heart disease including severe hypoplasia of posterior mitral valve leaflet.

Congenital heart defect is one of the major underlying cardiac conditions in patients with infective endocarditis. The incidence of endocarditis in adults with CHD has been reported to be 11 per 100 000 person‐years compared with 1.5‐6 per 100 000 patient‐years in general population. However, CHD‐associated mortality has decreased to 10% because of improvement in the diagnosis of infective endocarditis, antibiotic therapy, cardiac surgery, and interventional procedure.^1^ Herein, we aimed to describe a case of adult patient with a rare CHD who was complicated with infective endocarditis.

## 2 HISTORY OF PRESENTATION

A 23‐year‐old male patient was admitted to our hospital due to headache and decreased communication with others since 2 weeks ago. The patient also had history of generalized weakness, diarrhea, fever, and decreased appetite from 4 months ago. He did not complain of dyspnea, chest pain, dizziness, paresis, and paresthesia.

During physical examination at admission, the patient's vital signs were as follows: blood pressure :110/80 mm Hg, pulse rate: 120 beats per minute, respiratory rate: 18 per minute, temperature: 39.5°c, and O2 saturation: 90% in room air. The patient seemed ill and pail and had Broca aphasia. His skin was normal in inspection; no discoloration or lesion was found. In examination of thorax, we heard a diastolic murmur in precordium at aortic foci, holosystolic murmur in left parasternal border with decreased S1 intensity and S3 gallop. Lung sounds were clear except at upper part of right lung, and no rales were heard. Abdomen was soft with mild tenderness at left upper quadrant. His extremities were warm with no edema.

### 2.1 Past medical history

The patient was an active person up to 4 months ago and had no specific medical disorder and no history of trauma.

### 2.2 Differential diagnosis

According to patient's history and examination, differential diagnosis was central nervous system (CNS) infection or mass, infective endocarditis with emboli to CNS, and complicated gastrointestinal (GI) infection.

### 2.3 Investigations

**FIGURE 1:**
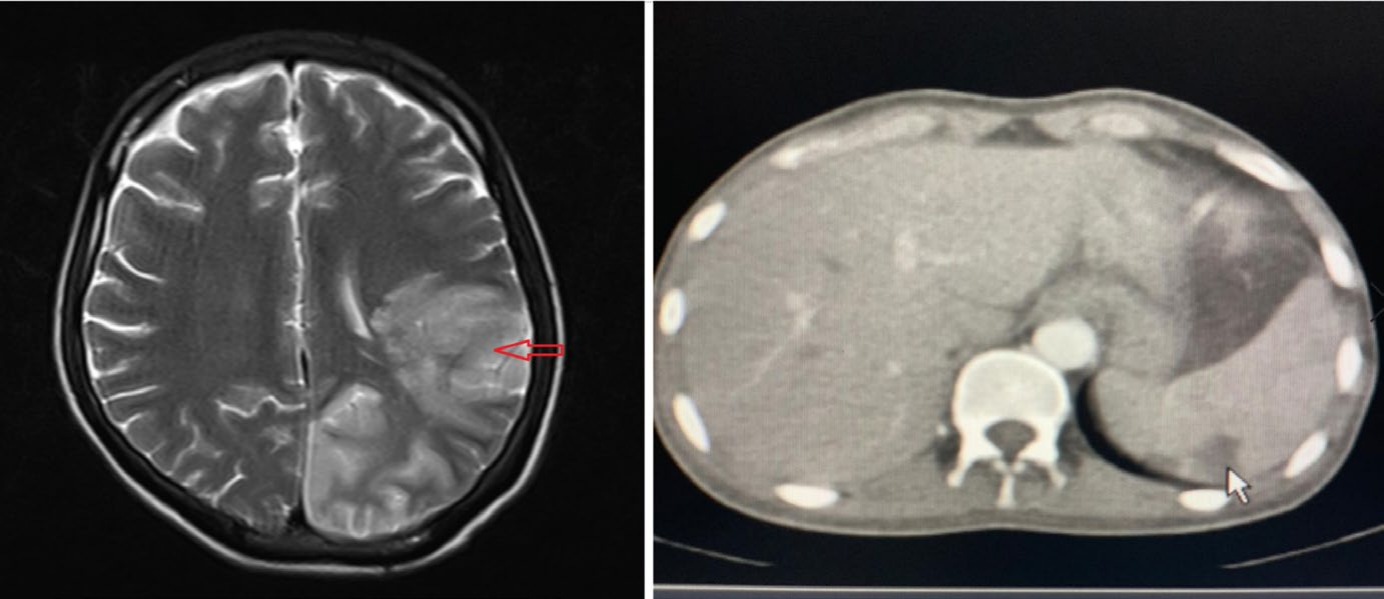
Left panel: Brain MRI showed hyperintense signals in parietal (red arrow) and occipital regions in T2 sequence in favor of infarction. Right panel: Abdominal CT showed hypodense lesions in lower pole of spleen (white arrow) suggestive of infarction

Initial laboratory findings showed anemia with hemoglobin: 7.6 g/dL, white blood cells: 10 000 per microliter, erythrocyte sedimentation rate: 48 mm/hr, and lactate dehydrogenase: 552 U/L. After that Brain Magnetic Resonance imaging (MRI) was done and showed hyperintense lesions in left parietal and occipital regions with cortical, subcortical and white matter involvement in T2 sequence in favor of infarction (Figure 1—left panel). Abdominal sonography and computed tomography (CT) showed free fluid in pelvic and hypodense peripheral lesions without vascularity (37*20 mm) in lower pole of spleen in favor of infarction (Figure 1—right panel).

**FIGURE 2:**
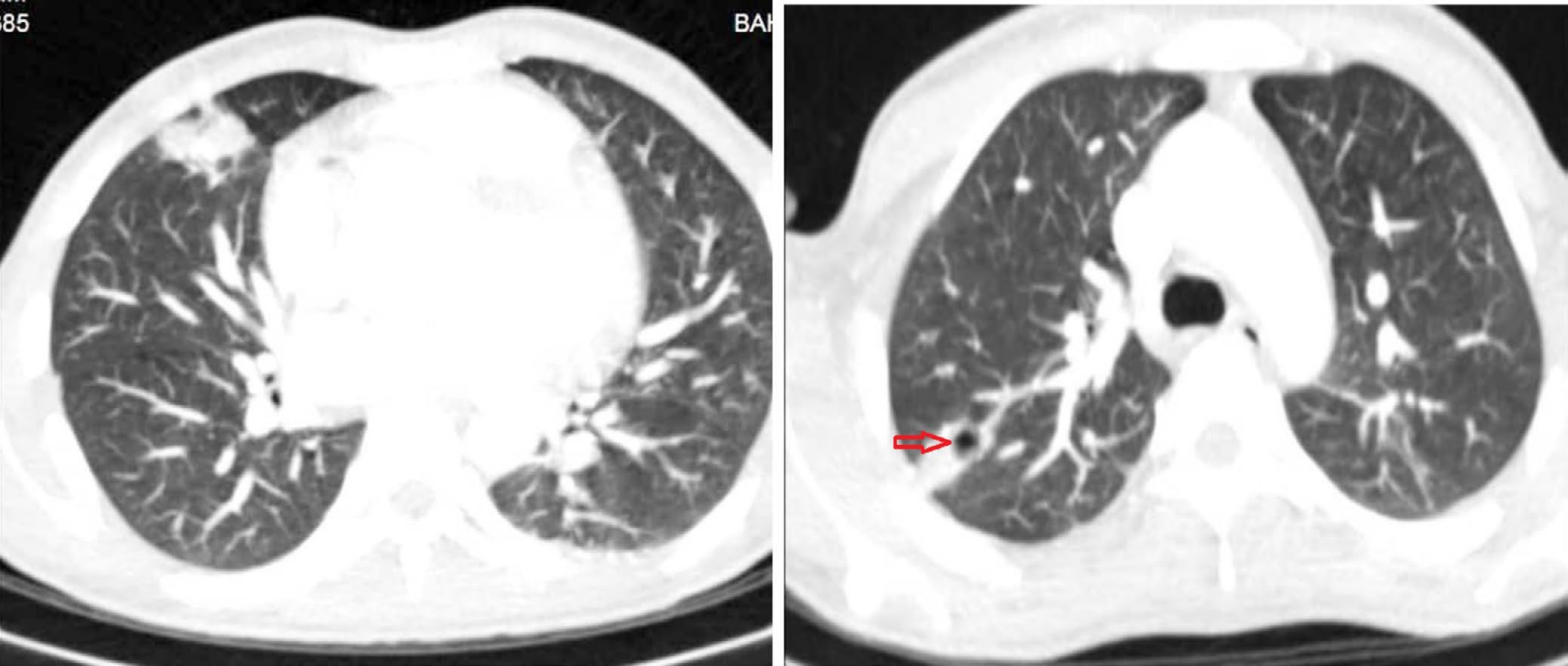
Chest CT of the patient that showed cardiomegaly with increased venous congestion (left) and a cavitary consolidation (red arrow) at upper lobe of right lung (right)

**FIGURE 3:**
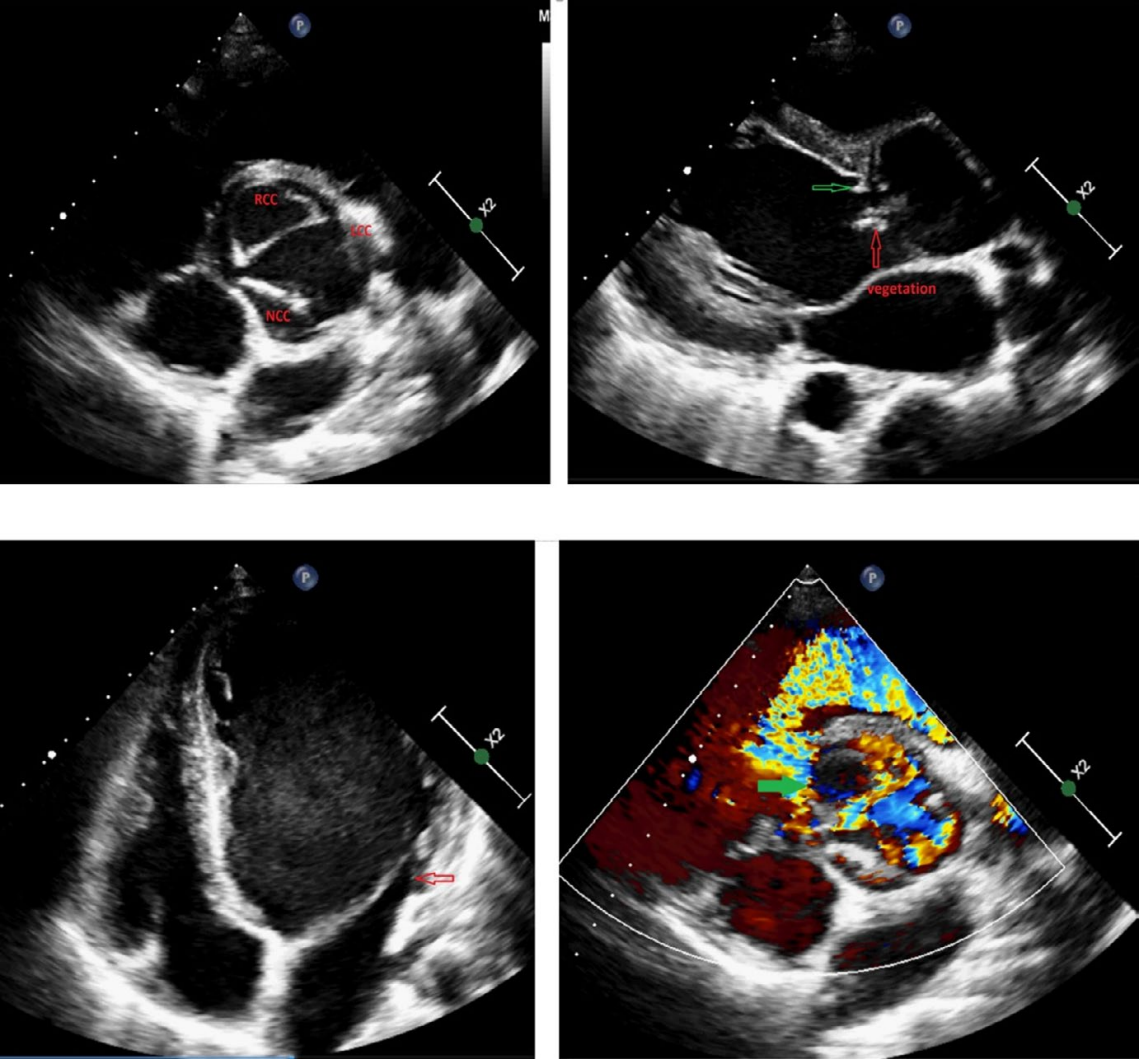
Top left: parasternal short‐axis view of aortic valve that is tricuspid with dissected RCC, top right: parasternal long‐axis view that showed small subaortic web (green arrow) and also vegetation on aortic valve (red arrow), bottom left: 4 chamber apical view showed left ventricular enlargement with severe hypoplasia of PMVL (red arrow), bottom right: parasternal short‐axis view of small perimembranous VSD (green arrow) with left to right shunt. RCC: right coronary cusp, LCC: left coronary cusp, NCC: noncoronary cusp, PMVL: posterior mitral valve leaflet, VSD: ventricular septal defect

Chest CT scan revealed prominent cardiomegaly with increased venous congestion and a cavitary consolidation at posterior segment of right upper lobe of lung in favor of septic emboli (Figure 2). The patient underwent transthoracic echocardiography for the evaluation of his murmur, emboli source, and vegetation, and it revealed severe left ventricular (LV) enlargement and dysfunction with LV ejection fraction about 20%. Small (5 mm) perimembranous ventricular septal defect (VSD) with left to right shunt was seen. There was a prominent muscle bundle in right ventricular outflow tract (RVOT; more than 2 cm distance from pulmonary valve) that resulted in dynamic turbulent flow at this site with peak gradient of 30 mm Hg suggestive of double chamber right ventricle (DCRV). Reversed doming of anterior mitral valve leaflet due to severe aortic regurgitation (AR) with severe hypoplasia of posterior mitral valve leaflet was seen in the assessment of mitral valve with mild mitral regurgitation (MR) and no significant stenosis. Aortic valve was tricuspid with dissected right coronary cusp and multiple mobile echo densities on left and right coronary cusps suggestive of vegetation that resulted in severe AR. There was also a small subaortic web with no significant stenosis. (Figure 3, Videos [Link], [Link], [Link], [Link]).

The patient was diagnosed infective endocarditis with multiple septic emboli and destructed aortic valve in context of congenital heart disease, and blood culture also was positive for Streptococcus Viridans.

### 2.4 Management

Patient received a complete course of antibiotic therapy with vancomycin and gentamycin according to antibiogram and also heart failure medication and then underwent open‐heart surgery of aortic valve replacement with resection of subaortic web and VSD closure successfully.

## 3 DISCUSSION

This was a case of an adult with multiple congenital heart disease including VSD, DCRV, subaortic web, and severe hypoplasia of PMVL that presented with complications of aortic valve endocarditis including multiple emboli to brain, lung, and spleen.

The association between infective endocarditis and acute aortic dissection is extremely rare;^2^, ^3^, ^4^ for example, Choe J et al reported a case of a 52‐year‐old man with sudden onset of dyspnea that brought to operation room with documented type A aortic dissection and severe AR. The operative findings show infective endocarditis involved the left coronary cusp of aortic valve and sinus of Valsalva, which causes dissection.^5^ Our patient had not any chest discomfort, and we think this localized aortic cusp dissection can be a complication of endocarditis, and otherwise, the painless aortic dissection is a very rare entity.^6^

Absence of PMVL is usually fatal in utero, while hypoplasia of it typically present in childhood with symptomatic mitral regurgitation ^7^ and its prevalence is estimated to be 1/8800.^8^ A few cases have been described in asymptomatic adults with hypoplasia of PMVL. One is a 35‐year‐old man with frequent premature ventricular contractions that had noncompaction LV and bicuspid aortic valve with severe hypoplastic PMVL and severe MR. Another case was an asymptomatic 21‐year‐old man with systolic murmur that in his echocardiography hypoplasia of PMVL was showed with mild MR.^9^ In this patient, association of these rare conditions was very grateful.

### 3.1 Follow‐up

After successful heart surgery and patient recovery, he was discharged home, and in 6‐month follow‐up, he was uneventful.

## CONFLICT OF INTEREST

None declared.

## AUTHOR CONTRIBUTIONS

FJ: wrote the initial draft, SAM: collected the patient's data, SJ: reviewed literatures, AS: edited the initial draft, KM: involved in concept, data collection, final edition of manuscript, and revision.

## ETHICAL APPROVAL

Written informed consent for publication of figures was obtained from patient.

## Supporting information

Video S1

Video S2

Video S3

Video S4

## Data Availability

Data are available on request due to privacy/ethical restrictions.
